# Dual-site theta stimulation modulates connectivity, but not sequence memory in older adults

**DOI:** 10.1093/braincomms/fcag153

**Published:** 2026-04-27

**Authors:** Nina M Ehrhardt, D Yorben Lodema, Robert Fleischmann, Ulrike Grittner, Dayana Hayek, Jevri Hanna, Robert Malinowski, Shu-Chen Li, Axel Thielscher, Agnes Flöel, Daria Antonenko

**Affiliations:** Department of Neurology, Universitätsmedizin Greifswald, Greifswald 17475, Germany; Department of Intensive Care Medicine and University Medical Center Utrecht Brain Center, University Medical Centre Utrecht, Utrecht, The Netherlands; Department of Psychiatry and University Medical Center Utrecht Brain Center, University Medical Centre Utrecht, Utrecht, The Netherlands; Department of Neurology, Universitätsmedizin Greifswald, Greifswald 17475, Germany; Berlin Institute of Health (BIH), Berlin 10178, Germany; Institute of Biometry and Clinical Epidemiology, Charité – Universitätsmedizin Berlin, Berlin 10117, Germany; Department of Neurology, Universitätsmedizin Greifswald, Greifswald 17475, Germany; Department of Neurology, Universitätsmedizin Greifswald, Greifswald 17475, Germany; University of Stuttgart, Stuttgart 70569, Germany; Department of Neurology, Universitätsmedizin Greifswald, Greifswald 17475, Germany; Chair of Lifespan Developmental Neuroscience, Faculty of Psychology, Technische Universität Dresden, Dresden 01062, Germany; Centre for Tactile Internet with Human-in-the-Loop, Technische Universität Dresden, Dresden 01062, Germany; Section for Magnetic Resonance, Department of Health Technology, Technical University of Denmark, Lyngby 2800 Kgs, Denmark; Danish Research Centre for Magnetic Resonance, Centre for Functional and Diagnostic Imaging and Research, Copenhagen University Hospital Amager and Hvidovre, Copenhagen 2650, Denmark; Department of Neurology, Universitätsmedizin Greifswald, Greifswald 17475, Germany; German Centre for Neurodegenerative Diseases (DZNE) Standort Greifswald, Greifswald 17489, Germany; Department of Neurology, Universitätsmedizin Greifswald, Greifswald 17475, Germany

**Keywords:** transcranial alternating stimulation, large-scale brain oscillations, ageing, non-invasive brain stimulation, episodic memory

## Abstract

Understanding and modulating memory functions in older adults continues to be a fundamental challenge for neuroscientific research. Given age-associated declines in long-range connectivity, network approaches targeting these connections are of particular interest. We investigated whether dual-site transcranial alternating current stimulation can modulate episodic (sequential) memory in cognitively healthy older adults (*N* = 44, aged 60–80 years). In a sham-controlled crossover design, participants received in-phase (0°) and anti-phase (180°) transcranial alternating current stimulation during a temporal order memory task, with a counterbalanced order of conditions. We computed source analysis-based weighted phase lag indices and corrected amplitude envelope correlation between left hemispheric fronto-parietal stimulation targets from resting-state electroencephalography to quantify modulation of functional connectivity, and conducted analyses of phase angles between these targets. No overall memory effects were observed in either active stimulation conditions, compared with sham. However, the results showed an interaction between memory modulation and age, indicating that the older the participants the higher the memory improvement in the anti-phase condition. Functional coupling increase was observed in both anti- and in-phase conditions as an elevated weighted phase lag indices theta change, compared with sham. No differences were observed for weighted phase lag indices in other frequency bands (alpha, beta) or for the corrected amplitude envelope correlation (theta, alpha, beta). Theta phase angle shifts were increased in the anti-phase compared with the sham condition. Further, in the anti-phase condition, the increase in theta connectivity was linked to age and memory improvement, indicating a potential mechanistic link between neurophysiological and cognitive outcomes. In sum, our findings suggest that dual-site anti-phase stimulation may increase functional connectivity in older adults with large interindividual variability in memory effects, warranting further investigation to optimize stimulation strategies.

## Teaser

Ehrhardt, Lodema *et al.* report no episodic (sequential) memory improvement through dual-site transcranial alternating stimulation in older adults. Source analysis-based weighted phase lag indices, quantified from resting-state electroencephalography before and after the stimulation, suggest a significant functional connectivity modulation between targeted areas which depends on individual memory improvement and age.

## Graphical Abstract

**Graphical Abstract:**
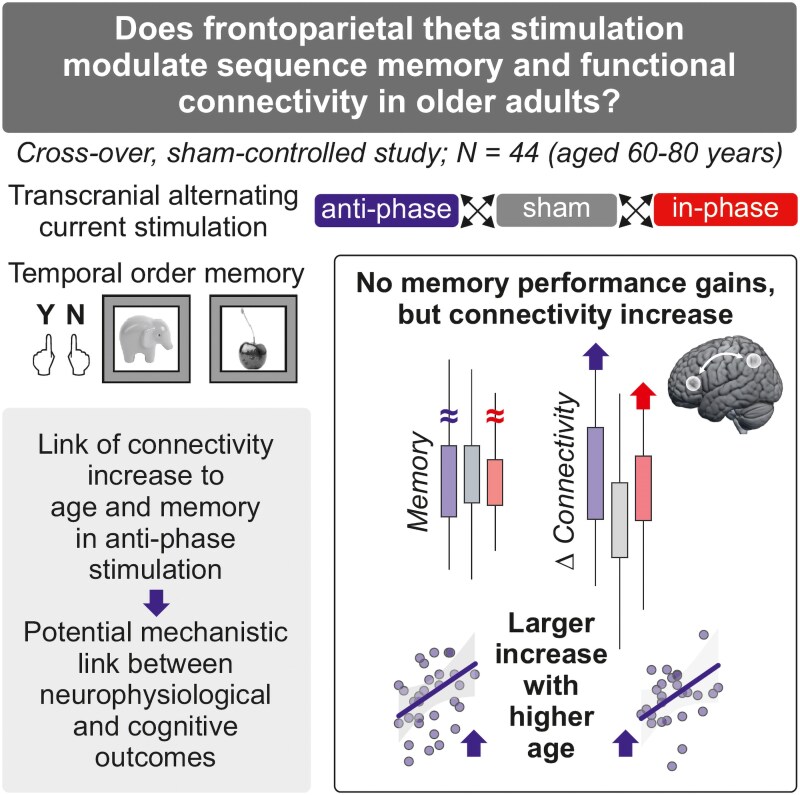

## Introduction

Cognitive functions depend on brain oscillatory activity and long-range connections between distant brain areas.^1,2^ Synchronously active neurons generate brain oscillations locally, which link information processing between brain regions.^3^ Age-associated cognitive decline may be due to dysfunctions on the network level,^4,5^ with long-range connections being especially affected by brain ageing.^6,7^ Frontoparietal phase synchrony in the theta band (4–8 Hz) is critical for optimal memory performance.^8,9^ Age-related decline in this synchrony has been implicated in memory impairments.^10^

Given this age-associated decline in long-range connectivity, network approaches targeting these connections are of particular interest. In transcranial alternating current stimulation (tACS), a current of sinusoidal waveform is applied with the direction of current flow changing at a specific frequency, to modulate amplitude, frequency or phase of brain oscillations, and consequently cognitive functions.^11,12^ Interfering with the synchrony oscillatory phases in the theta frequency band in distributed networks is possible with dual-site tACS^13,14^ and has potential to improve memory functions in ageing.^10,15,16^ A recent meta-analysis provides evidence for bidirectional effects when targeting two network hubs.^16^ Specifically, in-phase (i.e. 0° phase lag between the oscillatory current over two stimulation targets) stimulation appears to enhance behavioural outcomes, potentially via increased functional connectivity, in both young^17^ and older adults.^10^ In contrast, although the anti-phase (i.e. 180° phase lag between the oscillatory current over two stimulation targets) stimulation has been thought to impair performance as shown in few studies with young adults; the overall evidence is heterogeneous, with conflicting findings reported across studies, including reports of beneficial effects as well.^18^

Therefore, dual-site tACS may represent a particularly promising approach to enhance cognitive functioning by reinstating temporal coordination between distant brain regions.^10^ However, evidence for its effectiveness in older adults remains limited, and has thus far been restricted to studies on working memory enhancement.^10,19^ Whether episodic memory functions which are especially vulnerable to age-related decline,^20^ can be modulated by dual-site tACS targeting cortical network hubs remains unclear. No study to date has examined the impact of anti-phase tACS in older participants.

To address these questions, we conducted a randomized, sham-controlled, double-blind cross-over study in which in-phase (0°), anti-phase (180°), and sham 6-Hz theta-tACS were applied in a counterbalanced order to the left frontal and parietal regions of 44 cognitively healthy older adults during an episodic (sequential) memory task. The left hemisphere was targeted based on evidence from,^21^ which identified the left hippocampus as a critical region for temporal order memory. The prefrontal and parietal stimulation sites were selected based on their functional connectivity with the hippocampus and evidence that non-invasive brain stimulation of cortical regions can modulate activity in connected subcortical structures.^10,22,23^ In our previous work in young adults, we demonstrated that this frontoparietal electrode configuration increased oscillatory power in the hippocampus and its connectivity in individual with superior memory performance.^24^ Structural MRI was acquired to estimate individual electric field distributions and quantify field magnitudes, and electroencephalography (EEG) was recorded before and after stimulation to assess electrophysiological changes in connectivity. We focused on the two primary coupling modes, phase-coupling and amplitude coupling as quantified by the debiased weighted phase lag index (wPLI) and the corrected amplitude envelope correlation (AECc).^25,26^ Additionally, we examined the mean phase angle to assess the directionality and magnitude of phase shifts between stimulation targets.

We hypothesized that theta tACS could modulate memory performance and assumed there would be beneficial effects of the in-phase condition. While the anti-phase condition was assumed to impair performance, previous evidence yielded mixed results with findings pointing in all possible directions.^18^ We further aimed to assess whether field magnitudes were correlated to individual memory modulation. Resting-state EEG was conducted to assess changes in functional connectivity and to obtain potential mechanistic explanations for tACS effects.

## Materials and methods

### Study design

**Figure 1:**
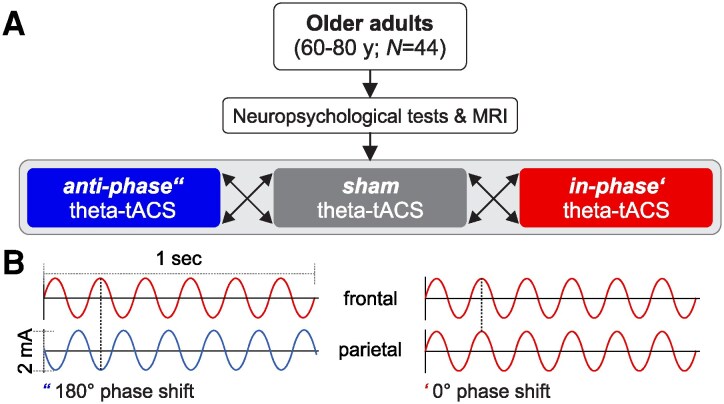
**Study design**. (A) Healthy older adults (60–80 years, *N* = 44) first underwent covariate assessment (neuropsychological tests, magnetic resonance imaging). Then, participants took part in three stimulation conditions (30-min in-phase theta- (6 Hz) tACS, 30-min anti-phase theta tACS, and sham (60 s in-phase) stimulation over frontal and parietal brain regions) in a counterbalanced order, separated by 1 week. (B) Illustration of anti-phase (180°) and in-phase (0°) phase shifts between frontal and parietal targets. mA, milliampere; MRI, magnetic resonance imaging; sec, seconds; tACS, transcranial alternating current stimulation; y, years.

The study was a double-blind, cross-over study with stimulation conditions administered in a counterbalanced order (Fig. 1). There were three stimulation conditions: in-phase stimulation (phase 0°), anti-phase stimulation (180° phase shift), and sham stimulation (60 s), applied in-phase as in previous studies.^10,14,17^ Participants were stratified by age with the cut-off at 70 years and randomly assigned to one of six possible orders of stimulation conditions (sham, in-phase, anti-phase; sham, anti-phase, in-phase; in-phase, sham, anti-phase; in-phase, anti-phase, sham; anti-phase, sham, in-phase; anti-phase, in-phase, sham) using block randomization. In the first session, a baseline assessment took place to assess demographics in a semi-structured interview, handedness using the Edinburgh Handedness Inventory,^27^ and to screen for depressive symptoms with the German short version of the Geriatric Depression Scale.^28^ Additionally, we conducted neuropsychological screening (Consortium to Establish a Registry for Alzheimer's Disease, CERAD-Plus Test Battery, https://www.memoryclinic.ch) in paper-pencil format to assess baseline cognitive functioning in a variety of cognitive domains, such as executive functions, processing speed, working memory, episodic memory, and to ensure cognitive functioning.^29^ Furthermore, participants performed a practice version of the sequence memory task.^21^ Next, we acquired MRI data at the Baltic Imaging Center (Institute of Radiology, University Medicine Greifswald, Germany). In the subsequent three sessions (separated by 1 week to avoid carry-over effects, Fig. 1A), participants received each of the three tACS conditions in a counterbalanced order during performance of a sequence memory task. Frontal and parietal stimulation was either applied anti-phase (180° phase shift), in-phase (phase 0°; Fig. 1B) or as sham stimulation, stopping after 60 s. Eyes-open resting-state EEG was recorded for six minutes before and after stimulation. To avoid unblinding through the ongoing EEG recording, one researcher interacted with the participant, and a second researcher recorded the EEG.^30^ To ensure blinding of the research staff interacting with participants, stimulation protocols for each participant and session were put in an envelope that was opened just before the stimulation by the researcher operating the EEG and stimulation device. An additional researcher, not aware of the applied stimulation protocol, interacted with the participant.^30^

### Participants

We included 44 right-handed cognitively healthy older adults (60–80 years) with normal or corrected-to-normal vision and intact hearing ability. Exclusion criteria were neurological or acute psychiatric diseases (e.g. stroke, epilepsy, traumatic brain injury, major depression, or anxiety disorder), impaired global cognition (Mini Mental State Examination^31^ score < 28) or memory (CERAD word list learning *z*-scores < −1.5^29^), and MRI or tACS contraindications (metal or electronic implants, pregnancy, large tattoos, claustrophobia). Furthermore, participants with poor memory performance close to chance levels (<60%) in the practice version of the sequence memory task during the screening session were not included in the study to ensure compliance with task instructions.^17,32,33^ The research complies with the Declaration of Helsinki and the ethics committee of University Medicine Greifswald gave their approval for the study (BB 021/19). Informed consent was obtained from all participants and participants received a compensation of 150€ for their participation. There was one drop-out after the first stimulation session due to skin irritation after the preparation of EEG electrodes, resulting in *N* = 43 [age in years, mean (SD) = 66.8 (4.3), female *n* = 20]. The baseline characteristics of the final sample are reported in Table 1.

**Table 1:** Baseline characteristics

	Mean/*n*	SD
Age (years)	66.8	4.3
Sex (female *n*)	20	
Education (years)	15.7	2.6
Edinburgh Handedness Inventory	95.0	9.6
Geriatric Depression Scale	0.8	1.0
Semantic Fluency	27.0	4.6
Boston Naming Test	14.9	0.3
Mini Mental State Examination	29.5	0.7
Word List Learning		
Total (max. 30)	21.6	2.7
Trial 1 (max. 10)	5.6	1.6
Trial 2 (max. 10)	7.4	1.4
Trial 3 (max. 10)	8.6	0.9
Word List Retrieval (max. 10)	7.6	1.3
Figure Copying (max. 11)	10.9	0.3
Figure Retrieval (max. 11)	10.5	0.9
Phonematic Fluency	15.7	5.2
Trail Making Test		
Part A (sec)	36.6	9.9
Part B (sec)	79.0	22.1

### Transcranial alternating current stimulation

**Figure 2:**
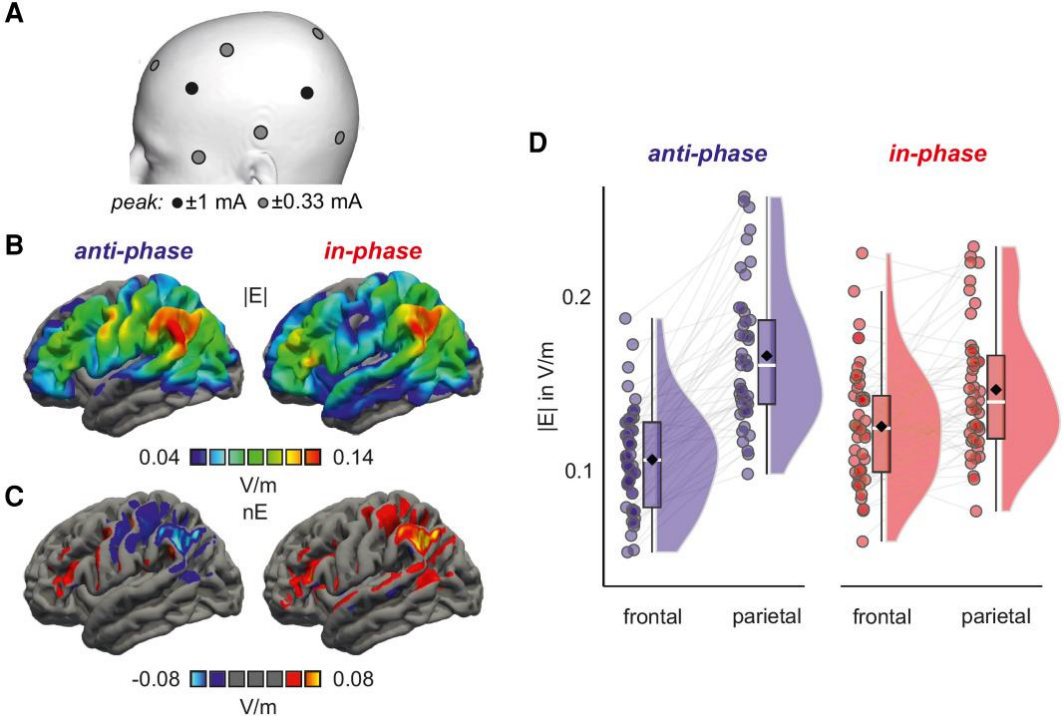
**Stimulation parameters**. (A) Electrode configurations for in- and anti-phase conditions. While in the in-phase condition, alternating current of 1 mA was applied with a 0° phase shift on the central (black) frontal and parietal targets, in the anti-phase condition, alternating current of 1 mA was applied with an 180° phase shift with the surrounding electrodes (grey) serving as return electrodes (0.33 mA each). (B) Average electric field magnitude (|E|) and (C) normal component of the field (nE) for anti-phase and in-phase tACS in V/m. (D) Electric field magnitudes (|E|) extracted from a sphere with a 10-mm radius around the peak frontal (−48, 26, 7) and parietal (−55, −48, 31) targets (*n* = 41). No statistical test was performed. Raincloud plots show individual data points with the mean (diamond), and a boxplot with the median (vertical line), 25th and 75th percentiles (lower and upper hinges), and 1.5*interquartile range (lower and upper whiskers). Individual data points represent field magnitudes (in V/m) for *n* = 41 participants. mA, milliampere. Note: A colour-blind friendly version of this figure using a viridis colormap is available in Supplementary Fig. 1.

We applied tACS for focal stimulation to target the fronto-parietal memory network using two battery-driven stimulators (DC Stimulator Plus; NeuroConn, neuroCare Group GmbH, Munich, Germany). Equalizer boxes (NeuroConn, neuroCare Group GmbH) split the bipolar channels of the stimulators in two 3 × 1 montages. Two central Ag/AgCl stimulation electrodes (diameter = 1 cm) were placed on the scalp of the left hemisphere above the frontal (in the centre of F3, F5, FC3, FC5) and parietal cortex (in the centre of P3, P5, CP3, CP5). Three surrounding electrodes for each central stimulation electrode were placed on the scalp equidistantly from the stimulation electrode (frontal electrodes: in the centre of F1, F3, AFz, and AF3, in the centre of C1, C3, FC1, FC3, below the middle of F7 and FT7; parietal electrodes: in the centre of CPz, CP1, Pz, P1, on PO7, in the centre of C5, T7, CP5, TP7, Fig. 2A). Stimulation electrodes were placed with the aim to (1) maximize electric field strength in these two brain regions with low relative stimulation of surrounding sites and (2) leave as many electrode positions as possible for EEG recording while at the same time assuring a constant distance of the surround electrodes. EEG caps were marked at the corresponding positions. Electrode positions were recorded for each participant using neuronavigation (Brainsight) after placement. Importantly, electrode position recordings were loaded for each participant from the previous sessions to ensure consistent placement. A current of 1 mA peak-to-baseline was applied for 30 min with 10 s ramp-up and ramp-down, respectively. Impedance of all electrodes was kept below 10 kΩ. A tACS frequency of 6 Hz was used, as in previous studies that have aimed to affect cognitive processes.^14,34^ Participants received each of the three tACS conditions in a counterbalanced order during performance of a sequence memory task. Frontal and parietal stimulation was either applied anti-phase (180° phase shift), in-phase (phase 0°) or as sham stimulation (phase 0°, stopping after 60 s, including the fade in/out intervals). To ensure blinding of the participants, a local anaesthetic (EMLA® cream, Aspen GmbH, Germany) was applied prior to stimulation. Blinding success and side-effects in response to tACS (e.g. tingling and/or burning sensations) were assessed using a side-effect questionnaire.^35^

### Electric field simulations

To visualize average distribution of electric fields (Fig. 2B and Fig. 2C), computational modelling was performed using SimNibs version 4.1 (simnibs.org) (Puonti *et al*., 2020; Thielscher *et al*., 2011). Head models were built using FreeSurfer v7 (Fischl, 2012) and charm, electric field simulations were conducted using default conductivity parameters implemented in the toolbox. A finite element mesh was generated from T1- and T2-weighted images, including representations of the scalp, skull, spongy bone, cerebrospinal fluid, grey matter, and white matter. To obtain regions-of-interest for EEG source analyses, the MNI coordinates of peak electric field magnitudes were extracted for the frontal and parietal stimulation ‘hot spots’ (peak frontal: −48, 26, 7, peak parietal: −55, −48, 31; Fig. 2D).

### Sequence memory task

**Figure 3:**
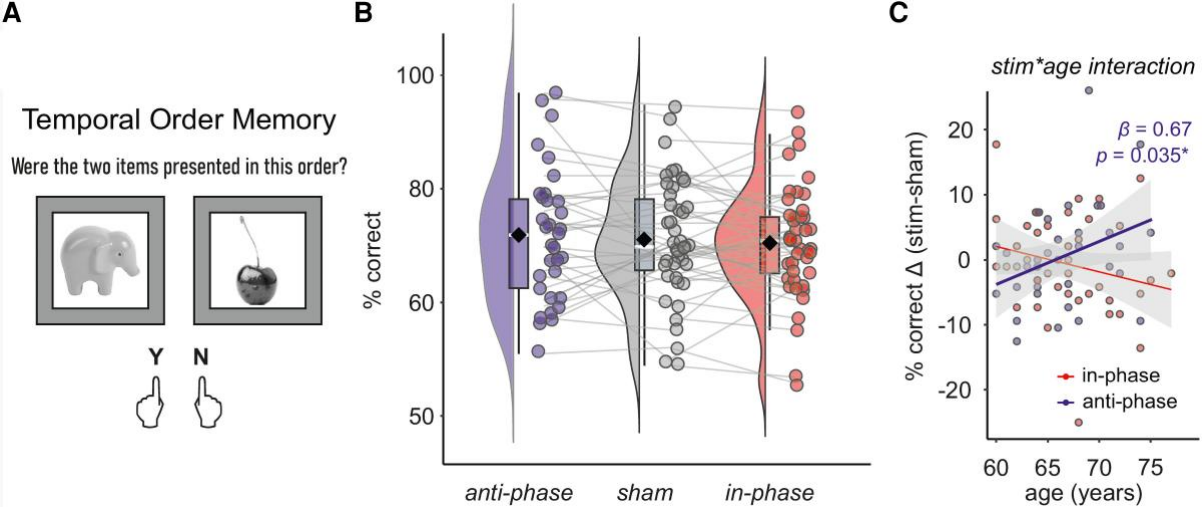
**Memory task performance**. (A) Example retrieval block item in the temporal order memory task. Participants had to indicate whether the order of two objects appears as in the preceding encoding phase by pressing left (yes) or right (no). (B) Memory performance (% correct) did not differ between anti-phase, sham, and in-phase stimulation (linear mixed model, pairwise comparisons anti-phase—sham: estimate [95%-CI] = 0.93 [−1.67, 3.52], *P* = 0.478, in-phase—sham: estimate [95%-CI] = −0.37 [−2.71, 1.98], *P* = 0.757). Raincloud plots display individual data points with the mean (diamond) and boxplots with the median (white line), 25th and 75th percentiles (lower and upper hinges), and 1.5 times the interquartile range (lower and upper whiskers). Individual data points represent memory performance (in % correct) for *n* = 43 participants. (C) Scatterplot to illustrate the stimulation condition × age interaction. Individual differences in memory performance (data points represent ‘stim minus sham’ difference of % correct in each individual) are displayed. In the anti-phase condition, we observed a significant interaction (linear mixed model, estimate [95%-CI] = 0.67 [0.06, 1.29], *P* = 0.035). Y, yes; N, no. # of participants: 43; # of observations: 119.

We used an adapted version of a sequence memory task^21^ as the main experimental paradigm. Participants saw two hundred forty grey-scale pictures of non-human objects without negative valence and not containing any text, presented sequentially in coloured frames. During encoding, each picture was on screen for 5000 ms, preceded by a fixation cross at the centre of the screen for 500 ms and followed by an inter-trial interval of 1000 ms. For a deeper encoding participants were encouraged to apply two memory strategies: (1) imagining the displayed object in the colour of the frame and indicating whether the colour fits the picture by button press; (2) imagining the object interacting with the previous object for sequence memory. After a list of five pictures, the colour of the frame changed. During retrieval after the presentation of four lists, two of the previously shown pictures were presented next to each other in a grey frame and participants indicated with a button press whether the pictures are presented in the right temporal order (see Fig. 3A for an example item). For each list, pictures from position 1 and 4 and 2 and 5 were presented in random order, resulting in eight retrieval trials per block. Correct and incorrect order was randomized with half of the trials in correct order. In total, 12 blocks of pictures were presented, resulting in a total number of 240 pictures with 12 retrieval blocks. We used three sets of pictures with equal number of items from each category and equal number of difficult items for the three stimulation conditions in counterbalanced order to minimize and account for learning effects. A pilot study with 12 young healthy adults (female = 10, age range: 19–29 years, mean = 23 years, SD = 3) confirmed that the three picture sets did not result in substantially different task performance measures (percent correct = 72.6, 95% CI = [67.0, 78.1]; 71.8, CI = [67.4, 76.1], 74.3, CI = [68.6, 80.0]; *F*(2) = 0.76, *P* = 0.48, *η*_p_^2^ = 0.065), indicating similar difficulty of parallel versions.

### Magnetic resonance imaging

A 3-Tesla scanner (Siemens Skyra) with a 32-channel head coil was used to acquire a T1-weighted (TR = 1690 ms, TE 2.52 ms, TI = 900 ms, 176 slices, 1.0 × 1.0 × 1.0 mm^3^, flip angle 9°, selective water excitation for fat suppression) and T2-weighted (TR = 12 770 ms, TE = 86.0 ms, 96 slices, 1.0 × 1.0 × 1.0 mm^3^, flip angle 111°) structural scans of the brain.

### EEG recording

EEG was recorded with a sampling rate of 1000 Hz using a 64-channel EEG-system (Brain Products GmbH, Germany). Sixty-two electrodes were placed on the scalp according to the international 10–20 system. Ground and reference electrodes were placed on electrode position FPz and on the nose tip, respectively. Two electrodes were placed next to the right and below the left eye to detect vertical and horizontal eye movements, as well as blinks. Impedances were kept below 5 kΩ with abrasive gel (Nuprep, Weaver and Company) for reference and EOG electrodes and Supervisc electrode gel for all electrodes to ensure good signal quality. The positions of the electrodes were sampled for each participant with neuronavigation (Brainsight) using the individual MRI scans of the participants. Following preparation of the electrodes, we recorded resting-state EEG for six minutes in which the participants fixated a cross on the computer screen. After the sequence memory paradigm with concurrent stimulation, we again recorded eyes-open resting-state EEG for 6 minutes with a fixation cross.

### EEG analysis

EEG analyses were conducted using MNE Python version 1.7.1.^36^

#### Preprocessing

First, data were down-sampled to 200 Hz and an offline band-pass filter of 1–30 Hz was applied. Then, channels containing no signal or substantial artefacts were detected using an automated non-ocular artefact removal (ANOAR)^37^ with a rejection threshold of *r* = 0.8. Next, data were decomposed using independent components analysis (number of components = 15). Components correlating (*r* ≥ 0.25 or *r* ≤ −0.25) with the EOG signal converted to bipolar vertical and horizontal EOG were rejected.^38^ Finally, data were epoched into trials of six seconds. To remove remaining high-amplitude artefacts, we applied a statistical rejection procedure: for each epoch, the maximum peak-to-peak amplitude across channels was computed, and epochs exceeding the individual mean by more than two standard deviations were excluded. To ensure reliability of the spectral estimates, sessions yielding fewer than 30 artefact-free epochs, amounting to three minutes of data, were excluded from further analysis. This threshold was chosen to exceed the minimum data length required for stable functional connectivity estimates (approximately 100 s^39,40^) by a conservative margin, while minimizing participant exclusion. However, all sessions in the final dataset retained at least 52 epochs, and thus no sessions were excluded based on this criterion.

#### Source analysis

To reconstruct sensor-level data in source space, individual head models were constructed from structural (T1-weighted) MRI scans using Freesurfer version 7.1.^41^ The head model was co-registered with the individual electrode positions sampled using Brainsight Neuronavigation. A distributed source space was created covering the entire cortical surface with a fixed spacing mechanism (oct6; indicating the resolution of the source space resulting in spacing of sources of approximately 4.9 mm). The forward solution was calculated in a boundary element model (BEM) with three layers (skin, outer skull, inner skull) and default conductivities (skin = 0.3 s/m, inner and outer skull = 0.006 S/m). Here, sources were not be allowed closer than 5 mm to the inner skull. Then, sources were reconstructed using weighted minimum-norm estimation (wMNE)^42^ with fixed dipole orientation. Source activity was extracted for two ROIs consisting of all dipoles within a 10 mm radius around the coordinates of the frontal and parietal peak efields (frontal: −48, 26, 7; parietal: −55, −48, 31) and summarized using the PCA Flip technique in MNE-Python, which combines the phase information of the multiple sources within a given ROI better than a simple mean. Seven participants could not be included in source analysis due to technical errors creating the individual head models. The errors occurred during BEM model construction in MNE-Python, when the inner and outer skull surfaces were improperly nested or positioned with insufficient separation, preventing the creation of valid forward models for source localization. Potential causes for these segmentation errors include low tissue contrast between skull and brain in the T1-weighted images, motion artefacts during MRI acquisition, anatomical variations, or suboptimal image quality in specific head regions. We did not attempt manual correction of these surfaces, as this would introduce additional variability and potential bias into the source reconstruction pipeline.

#### Connectivity analysis

Only participants with at least 75% remaining trials were included in the analyses (see Supplementary Table 1 for an overview of EEG data after preprocessing). We used the extracted time courses from source space ROIs to calculate connectivity estimates between the frontal and parietal ROIs using the spectral_connectivity_epochs function from the MNE-connectivity toolbox version 0.7.0. To assess phase-coupling, we computed the debiased weighted phase-lag index (wPLI; method='wpli2_debiased’) (wPLI).^43^ To assess amplitude-coupling, we computed the amplitude envelope correlation (AEC). The analytic signal was obtained via the Hilbert transform (scipy.signal.hilbert), and to correct for signal leakage, signals were orthogonalized prior to envelope calculation using a symmetric approach. Here, the signal of one ROI is orthogonalized with respect to the other and vice versa, and the resulting correlations were averaged, resulting in a corrected AEC (AECc).^44^ Finally, to quantify the phase shift between stimulation targets, we calculated the mean phase angle. This angle was derived by computing the complex coherency (method='cohy’), averaging the complex values across the frequency band, and extracting the angle from the resulting mean complex vector. This metric reflects the dominant temporal relationship between the two regions of interest, indicating the magnitude and shift of the oscillatory lag (i.e. which region leads or lags compared with the other). Calculating phase angles in source space does not allow a precise determination of the physical directionality of reconstructed electrical fields. Because EEG source reconstruction solves an ill-posed inverse problem with infinitely many mathematically plausible solutions, the absolute orientation (i.e. sign) of the reconstructed dipole or field vector is not uniquely determined. This sign ambiguity does not reflect random variation but a fundamental property of the inverse solution; if uncorrected, it precludes interpreting vector directions as true current flow. Importantly, a global sign flip of one source shifts its phase by π, which means that phase differences can only be resolved modulo π rather than 2π. As a result, full phase angles between signals cannot be reconstructively estimated, effectively restricting interpretable phase-difference estimates to a ± 90° range (i.e. modulo π). Nonetheless, this allowed us to assess whether the different stimulation conditions successfully induced the targeted shifts in synchronization dynamics. All measures were calculated for theta (4–8 Hz), alpha (8–12 Hz), and beta (12–30 Hz) frequency bands and averaged over epochs for each participant.

### Statistical analysis

Statistical analysis was conducted with R software version 4.4.1 (http://www.R-project.org) and packages lme4,^45^ blme,^46^ and emmeans.^47^ To estimate memory performance differences between sham and active tACS stimulations in overall response accuracy (% correct answers) we used linear mixed models with random intercepts for individuals^48^ and fixed effects for stimulation condition (‘in-phase’, ‘sham’, ‘anti-phase’). Individual order of stimulation condition, session number, age, and baseline cognitive performance (% correct answers in the practice version of the sequence memory task) were included as covariates. Interaction terms for stimulation condition and age, and stimulation condition and baseline performance were included. To estimate differences between sham and active tACS stimulations in connectivity measures and phase angle changes (post minus pre), linear mixed model with random intercepts for individuals^48^ and stimulation condition (‘in-phase’, ‘sham’, ‘anti-phase’) for the difference between post and pre stimulation was tested, including age as covariate. Additionally, an interaction term for stimulation condition and age was included to test for differences of the stimulation effects with age. Model assumptions were systematically evaluated, and model fit metrics are reported in Supplementary Table 2. All models met assumptions without substantial violations. We report mean differences between in-phase or anti-phase tACS and sham and 95% confidence intervals (CI) based on marginal mean estimates. Differences in side-effects (occurrence of side-effects: yes/no) between stimulation conditions and sham was analysed with binary logistic mixed-model analysis with random intercept for individuals and adjusted for age and sex. The blinding success was evaluated using a binary logistic mixed model with random intercept for individuals and the actual stimulation (active or sham) as binary outcome and the guessing (active, indifferent, sham) as independent variable. Odds ratios and 95% CI as well as marginal probabilities are reported.

## Results

### No overall effect of left frontoparietal theta-tACS on sequence memory performance in older adults

Memory performance (% correct responses) did not differ between stimulation conditions (estimated marginal means [95%-CI]: 71.8 [68.6, 75.1] for anti-phase, 70.9 [67.9, 74.0] for sham, 70.6 [67.5, 73.6] for in-phase; pairwise comparisons anti-phase—sham: estimate [95%-CI] = 0.93 [−1.67, 3.52], *P* = 0.478, in-phase—sham: estimate [95%-CI] = -0.37 [−2.71, 1.98], *P* = 0.757, Fig. 3B). The model explained 77% of total variance (conditional *R*^2^ = 0.77), with fixed effects accounting for 26% (marginal *R*^2^ = 0.26). An interaction of stimulation with age in the anti-phase condition indicated a dependency of individual improvement on age (estimate [95%-CI] = 0.67 [0.06, 1.29], *P* = 0.035, Fig. 3C). Estimated marginal means showed that anti-phase stimulation increased memory performance in older participants (effect of stimulation condition (anti-phase—sham) at 72.5 years = 4.79, 95%-CI = 0.30, 9.27, *P* = 0.037; effect at 62.5 years = −1.94, 95%-CI = −5.61, 1.72, *P* = 0.295). Individual electric field magnitudes in the stimulation targets did not correlate to individual memory improvement (all |rho|'s ≤ 0.26, all *p*'s ≥ 0.12, Supplementary Fig. 1).

### Increased functional connectivity between target sites through anti-phase and in-phase theta-tACS in older adults

**Figure 4:**
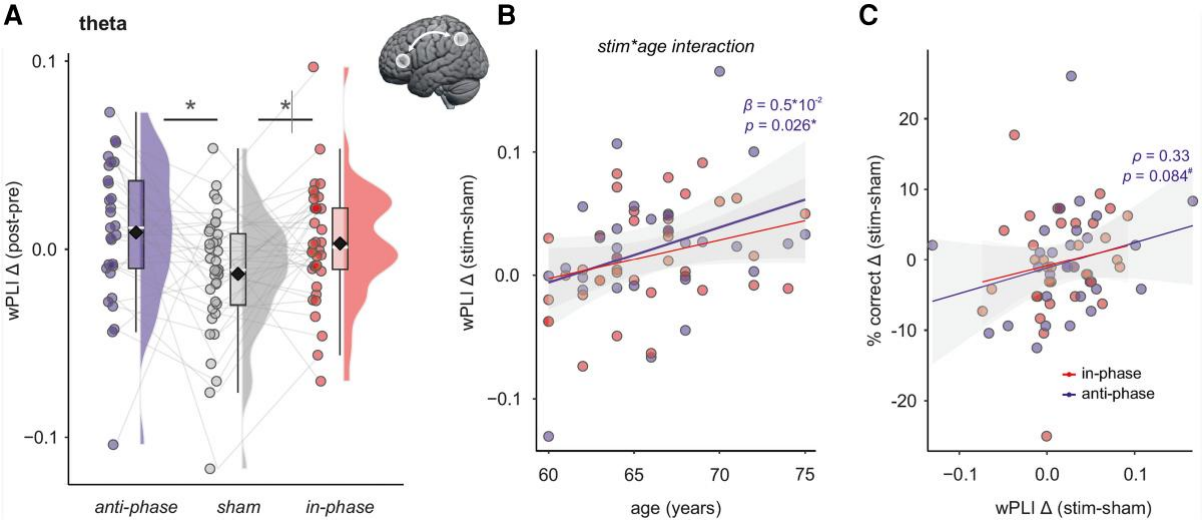
**Connectivity**. (A) Fronto-parietal theta connectivity was higher after anti-phase and in-phase stimulation compared sham (linear mixed model, pairwise comparisons: anti-phase—sham: estimate [95%-CI] = 2.18 × 10^−2^ [0.56, 3.80], *P* = 0.009, in-phase—sham: 1.66 × 10^−2^ [0.14, 3.18], *P* = 0.033). Raincloud plots show individual data points with the mean (diamond), and boxplots with median (white line), 25th and 75th percentiles (lower and upper hinges), and 1.5*interquartile range (lower and upper whiskers). Individual data points represent connectivity (as debiased wPLI) for *n* = 36 participants. (B) Scatterplot to illustrate the stimulation condition × age interaction. Individual differences in wPLI change (stim minus sham) are displayed. In the anti-phase condition, we observed a significant interaction (linear mixed model, estimate = 0.5 × 10^−2^, 95% CI: [0.07, 0.88], *P* = 0.026). (C) Scatterplot to illustrate the relationship between individual wPLI change (data points represent the difference between post and pre timepoints, subtracting ‘stim minus sham’ in each individual) and modulation of memory. Individual difference in memory performance (data points represent ‘stim minus sham’ difference of % correct in each individual) are displayed. Connectivity and memory modulation were correlated in the anti-phase (Spearman's rho = 0.33, *P* = 0.084), but not in the in-phase condition (Spearman's rho = 0.26, *P* = 0.130). wPLI = debiased weighted phase-lag index; # of participants: 36; # of observations: 101; *P < 0.05; ^#^*P* < 0.10.

Functional connectivity as quantified by the wPLI showed an increase in anti-phase and in in-phase conditions, compared with sham (estimated marginal means [95%-CI] for post-pre differences: 0.84 × 10^−2^ [−0.40, 2.09] for anti-phase, −1.34 × 10^−2^ [−2.45, 0.22] for sham, 0.32 × 10^−2^ [−0.79, 1.43] for in-phase; pairwise comparisons: anti-phase—sham: estimate [95%-CI] = 2.18 × 10^−2^ [0.56, 3.80], *P* = 0.009, in-phase—sham: 1.66 × 10^−2^ [0.14, 3.18], *P* = 0.033, Fig. 4A). The model explained 20% of total variance (conditional R^2^ = 0.20), with fixed effects accounting for 13% (marginal R^2^ = 0.13). An interaction of stimulation with age in the anti-phase condition indicated a dependency of individual wPLI increase on age (estimate = 0.47 × 10^−2^, 95% CI: [0.07, 0.88], *P* = 0.026, Fig. 4B). Estimated marginal means showed that anti-phase stimulation increased memory performance in older participants (effect of stimulation condition (anti-phase—sham) at 72.5 years = 5.24 × 10^−2^, 95%-CI = 2.14, 8.35, *P* = 0.001; effect at 62.5 years = 0.50, 95%-CI = −1.70, 2.71, *P* = 0.651).

Exploration of the relationship between individual theta connectivity increase and memory modulation showed a trend for a positive correlation in the anti-phase condition (Fig. 4C), suggesting that individuals for whom the stimulation increased functional connectivity may exhibit a larger cognitive benefit (Spearman's rho = 0.33, *P* = 0.084).

Change in wPLI was specific to the theta band, with no differences between conditions observed for alpha or beta bands (Supplementary Fig. 2 and Supplementary Table 3 for estimated marginal means and pairwise comparisons).

Comparison of fronto-parietal amplitude-envelope correlation (AECc) did not show differences between conditions for theta, alpha, or beta bands (Supplementary Fig. 3 and Supplementary Table 4 for estimated marginal means and pairwise comparisons).

**Figure 5:**
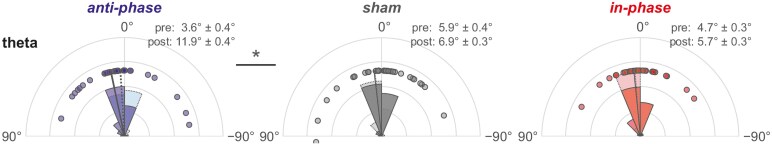
**Theta phase angles**. Phase angle distributions (degrees) for theta frequency shift between stimulation targets and individual difference values between stimulation and sham conditions (blue dots). Histograms for pre (light blue) with mean vector (dashed line), and post (dark blue) with mean vector (solid line). In the anti-phase condition, we observed a significant stimulation condition × age interaction (linear mixed model, estimate = 3.85; 95% CI: [0.47, 7.24], *P* = 0.029). # of participants: 36; # of observations: 101. *P < 0.05.

Descriptive analysis of the shift of oscillatory activity between stimulation targets did not provide evidence for an overall difference between stimulation conditions (estimated marginal means [95%-CI]: 6.83 [−3.68, 17.3] for anti-phase, 1.46 [−7.94, 10.9] for sham, 1.21 [−8.19, 10.6] for in-phase; pairwise comparisons anti-phase—sham: estimate [95%-CI] = 5.36 [−8.12, 18.8], *P* = 0.430, in-phase—sham: estimate [95%-CI] = −0.25 [−12.9, 12.4], *P* = 0.969, Fig. 5). The model explained 17% of total variance (conditional R^2^ = 0.17), with fixed effects accounting for 7% (marginal *R*^2^ = 0.07). An interaction of stimulation with age in the anti-phase condition indicated a dependency of individual modulation on age (estimate = 3.85, 95% CI: [0.47, 7.24], *P* = 0.029). Estimated marginal means showed that anti-phase stimulation increased phase angle shift towards positive values in old-older participants of our cohort (effect of stimulation condition (anti-phase—sham) at 72.5 years = 30.25, 95%-CI = 4.38, 56.12, *P* = 0.023; effect at 62.5 years = −8.27, 95%-CI = −26.6, 10.1, *P* = 0.372). All other comparisons across frequency bands were non-significant (all *P*'s > 0.2, Supplementary Fig. 4).

### Adverse effects and blinding

Most participants reported expected side effects, such as itching, warmth, and burning under the electrode, with mild (44.5%) to moderate (38.7%) intensity, or no side effects (11.8%), as reported in Supplementary Table 5. There was no substantial difference between active stimulation conditions and sham (anti-phase: odds ratio = 2.85, 95%-CI [0.19, 43.5], *P* = 0.451; in-phase: odds ratio = 0.54, 95%-CI [0.06, 4.78], *P* = 0.579). In one-third of the situations, participants correctly guessed that they had received active stimulation, and in 3.4% of the situations, participants correctly guessed that they had received sham stimulation (Supplementary Table 6). The odds ratio of correctly guessing an active condition compared with wrongly judging an active condition as sham was 4.32 (95% CI [0.70, 26.56], *P* = 0.114). The odds ratio of an indifferent guessing if actually an active condition compared with wrongly judging an active condition as sham was 3.45 (95%-CI [0.56, 21.26], *P* = 0.182), indicating successful blinding.

## Discussion

In this double-blind, sham-controlled crossover study, we aimed to modulate episodic (sequential) memory through theta-frequency, dual-site fronto-parietal transcranial alternating current stimulation in healthy older adults. We observed no overall effect on memory performance, but found that memory improvement in the anti-phase condition was dependent on age. Both in-phase and anti-phase fronto-parietal theta tACS increased functional connectivity between the stimulation targets. Connectivity increases following anti-phase stimulation compared with sham were more pronounced in older participants and correlated with memory improvement. Descriptive analyses of phase angle shifts suggested effective modulation in the anti-phase condition.

### Memory performance

Our study found no overall effect of either in-phase or anti-phase 30-minute theta tACS on episodic memory performance in older adults. These findings contribute to a growing body of evidence highlighting substantial inter-individual variability in responses to non-invasive brain stimulation,^12,49^ including reports of tACS ineffectiveness.^50^ Consistent with our recent meta-analysis demonstrating on average 20% greater variability in response to transcranial direct current stimulation in older compared with young adults,^51^ we observed considerable performance differences between older participants in the current study, with individual stimulation response differences ranging from approximately −20% to 20% (performance in active minus sham condition).

Given the complex nature of episodic memory—encompassing encoding, consolidation, and retrieval—stimulation effects on behavioural performance may be specific to particular memory subtasks. Future research should explore blocked stimulation protocols tailored to the cognitive state and task demands.^16,17,52^

Previous dual-site tACS studies primarily focused on working memory, reporting performance improvements through rhythmic cortical synchronization in the theta frequency band.^10,14,16,17^ However, findings remain inconsistent.^18^ Temporal order memory, an episodic memory function mediated by subcortical hippocampal networks,^21^ relies on a widespread network involving the hippocampus, medial temporal lobe, and various cortical regions. Stimulating the frontoparietal network alone may be insufficient to significantly enhance this memory function.^18^ While the rationale for stimulating connected cortical sites using tACS or tDCS appears compelling, emerging stimulation approaches designed to target deep brain structures non-invasively may prove more effective.^53,54^

### Age-dependent effects

Previous studies have predominantly observed beneficial memory effects of in-phase stimulation. In contrast, findings on anti-phase stimulation have been more heterogeneous, with studies reporting working memory improvements, impairments, or null effects for a review, see.^18^ Our observation of an interaction between memory effects and age in the anti-phase condition indicated that older participants showed greater memory benefits. Although anti-phase stimulation has decreased performance in some studies with young adults, firm conclusions are not yet possible and require further investigation.^18^ Notably, no studies have yet examined the effects of anti-phase stimulation specifically in older adults.

Similarly, a recent study^55^ demonstrated that frontal theta tACS enhanced associative memory only in the oldest participants, possibly because age-related declines in frontal neuron efficiency increased susceptibility to tACS entrainment. This potential benefit aligns with theoretical frameworks proposing that phase shifts between frontal and parietal sites beyond 0° may reflect direct connectivity between these regions and thereby enhance memory function.^8,56^ In older adults, anti-phase stimulation may promote interregional communication and re-establish functional co-activity, for instance, between cortical sites and hippocampal structures.^23,57^ Prior studies have also suggested that the mechanisms underlying stimulation effects may differ between older and younger participants.^58-60^ Together with our findings, these observations underscore that age is a crucial factor modulating the effects of non-invasive brain stimulation.^58,61,62^

Our results extend previous evidence by demonstrating that stimulation effects differ between age groups (young versus old) and become even more pronounced with advancing age within the older adult population. This may reflect ongoing age-related decline in metabolite function, progressive atrophy of grey and white matter, and alterations in the dynamics of large-scale brain networks,^63-65^ which could underlie distinct stimulation responses—a hypothesis warranting evaluation through studies incorporating broader age ranges.

### Functional connectivity

We assessed functional connectivity between stimulation targets using a peak electric field modelled on participants’ individual brain anatomy, quantifying the weighted phase lag index (wPLI) and the amplitude envelope correlation corrected for signal leakage (AECc).^25^ We observed increased wPLI for fronto-parietal theta activity following tACS, indicating that theta tACS enhanced fronto-parietal theta connectivity for both in-phase and anti-phase stimulation conditions. The effect of increased connectivity following in-phase stimulation is consistent with previous EEG and fMRI findings.^10,17,66,67^ For anti-phase tACS, Reinhart^66^ reported decreased connectivity measures, whereas other studies have found increased connectivity or no change following anti-phase stimulation.^17,67,68^ Increased wPLI following anti-phase stimulation may arise because connectivity measures quantify the degree of consistency, and thus coupling strength, between two signals.^68-70^ To examine the underlying phase relationships, we analysed angle shifts derived from complex coherency in the theta frequency band, revealing a larger phase shift in the anti-phase condition than in the sham condition, with age-dependent differences. While exploratory, these findings provide initial evidence that dual-site tACS can enhance functional connectivity in healthy older adults, possibly through reinstating beneficial phase lags critical for network functioning. A deviation from a 0° phase offset may be functionally advantageous, because synaptic delays and axonal conduction times across long-range corticocortical pathways can sum to several milliseconds, typically ∼1 ms per synapse plus ∼0.1 ms/mm of axonal path length, yielding total delays of up to ∼10 ms.^71^ A phase shift of 10° in the theta band accommodates these physiological delays: at 6 Hz, one cycle corresponds to 200 ms, so a 10° shift equals ∼6 ms. Thus, non-zero phase offsets may be biologically necessary to align presynaptic spike arrival with postsynaptic excitability windows and thereby promote spike-timing–dependent plasticity in long-range connections. In contrast, perfect 0° synchrony between distant regions may actually reduce the likelihood of effective spike-timing relationships.^72^

Notably, older adults demonstrated greater connectivity modulation, and this connectivity modulation was associated with memory effects. Interestingly, we observed a trend towards a moderate correlation between anti-phase stimulation effects on memory performance and theta connectivity, but no such association for in-phase stimulation effects. Although not reaching statistical significance, this trend suggests that increased fronto-parietal connectivity induced by anti-phase stimulation may represent a relevant mechanism underlying memory improvement in older adults.

In contrast to in-phase stimulation, anti-phase stimulation increases the phase lag between stimulated brain areas.^68^ Consequently, the beneficial effects of anti-phase stimulation align with the hypothesis that direct transmission between two brain areas results in phase lags due to conduction delays.^8,70^ Although not formally investigated in this study, one might tentatively hypothesize that, while both stimulation conditions increase connectivity measures, only anti-phase stimulation enhances memory performance by enhancing coupling at a specific, functionally optimal phase lag. This phase lag may become more pronounced with increasing age due to less efficient signal transmission caused by age-associated impairment of microstructural integrity in white matter pathways,^73^ potentially explaining the larger memory effect observed in older participants.

Future studies should investigate the specific phase shift required for optimal episodic memory performance and test its causal relevance and specificity using stimulation protocols with systematically varied phase shifts^74,75^ or by dynamically adjusting stimulation phase to ongoing endogenous phase shifts using closed-loop protocols.^76^

### Strengths and limitations

The EEG measures were obtained using an offline design, reflecting changes from pre- to post-stimulation. While online recordings could elucidate processes occurring during stimulation, EEG recorded during ongoing tACS contains substantial stimulation artefacts, making interpretation extremely challenging.^75,77^ However, resting-state EEG recorded before and after stimulation may reflect entrainment effects that outlast the stimulation period or neuroplastic changes induced by the intervention.^75^ Additionally, although exploratory, the selective correlation between behavioural and neurophysiological stimulation effects provides further support for the utility of pre- and post-stimulation resting-state EEG recordings in elucidating potential mechanisms of tACS—findings that should be validated in future studies employing appropriate algorithms to remove stimulation artefacts from online EEG.

It remains unclear whether stimulation at individual peak frequency is optimal, or whether there exists a universally optimal frequency for the cognitive function being targeted.^75,78^ Additionally, the uncertain reliability of peak frequency estimates,^79^ and the limited range of individual frequencies observed in older adults currently restrict meaningful individualization of stimulation parameters.^19^ Previous studies have suggested beneficial effects when a uniform stimulation frequency is applied across all participants.^78,80^ Therefore, given the current challenges regarding individual frequency determination and existing evidence of oscillatory entrainment through fixed theta tACS at 6 Hz,^14,17,34,66,81^ we elected to investigate the modulation of episodic memory networks using an empirically determined fixed frequency appropriate for the studied age cohort.

## Conclusions

In summary, tACS effects have demonstrated considerable variability across previous work and in the present study. Mechanisms underlying these inconsistent results may include differences in stimulation parameters (frequency, intensity, duration, electrode placement), individual neuroanatomical variability, baseline cognitive status, and the specific cognitive processes being targeted.^18^ This heterogeneity highlights the critical need for future research to elucidate the structural organization and temporal dynamics of networks underlying successful episodic memory formation in specific populations and across individuals, thereby enabling better tailoring of stimulation protocols to the requirements of individual neural networks and optimization of stimulation parameters.

To improve the efficacy of tACS for episodic memory enhancement, particularly in older adults, we recommend several avenues for future investigation: First, more targeted stimulation of specific network nodes based on individual connectivity patterns could improve intervention specificity.^23^ Second, development of algorithms to personalize tACS parameters (frequency, intensity, duration) according to individual brain states and cognitive profiles may enhance treatment response.^18,49^ Third, exploration of deep stimulation methods such as temporal interference stimulation to directly target subcortical structures,^53,54^ potentially in combination with cognitive training, may overcome the limitations of cortical stimulation approaches.^16,82^

Stimulating the fronto-parietal theta network with tACS holds potential to improve episodic memory performance in healthy older adults by reinstating optimal network function during ageing. Our results indicate that: (1) there is no evidence for improvement of episodic memory performance through fronto-parietal in-phase tACS in healthy older adults, (2) anti-phase tACS may improve memory performance compared with sham stimulation, with larger effects in older individuals, (3) both in-phase and anti-phase stimulation can modulate long-range functional connectivity, and (4) the beneficial effect of anti-phase tACS on episodic memory performance may be mediated through modulation of network connectivity.

However, substantially more research is needed to determine optimal phase shifts for memory network communication, which will require larger cohorts and broader age ranges. Future studies should help elucidate optimal oscillatory phase lags across the lifespan and determine their functional specificity. Additionally, tACS studies systematically varying phase relationships between 0° and 180°could help establish the causal mechanisms underlying the beneficial effects of specific phase shifts.^74^ This approach could ultimately advance dual-site tACS as a viable intervention for age-related cognitive decline and, potentially, more pronounced cognitive impairment in neurodegenerative diseases.

## Supplementary Material

fcag153_Supplementary_Data

## Data Availability

The data that support the findings of this study are available from the corresponding author (D.A.) upon reasonable request. The code used in this paper is available in via the Open Science Framework (OSF): https://osf.io/u854p.
