# Supplementary material for: Dual-site theta stimulation modulates connectivity, but not sequence memory in older adults
Source: Brain Commun. 2026 Apr 27;8(3):fcag153. doi: 10.1093/braincomms/fcag153 (PMC13166891; doi:10.1093/braincomms/fcag153)
Supplement: fcag153_Supplementary_Data [file fcag153_supplementary_data.docx]

# **Supplementary Material**


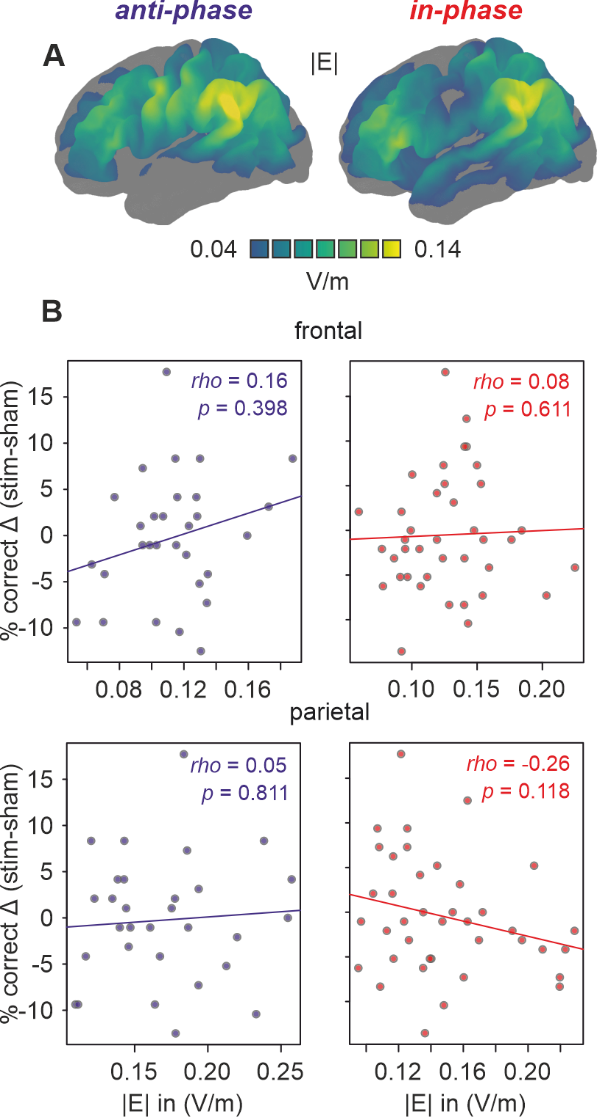


**Supplementary Figure 1.** Electric fields. (A) Color-blind friendly version of main **Figure 2**: average electric field magnitude (|E|) for anti-phase and in-phase tACS in V/m. (B) Scatterplots for correlations of individual field magnitudes (individual data points represent electric field magnitudes (|E| in V/m) extracted from a sphere with a 10-mm radius around the peak frontal (-48, 26, 7) and parietal (-55, -48, 31) in targets and memory modulation. Individual electric field magnitudes in the stimulation targets did not correlate to individual memory improvement (Spearman’s correlation coefficients, all |rho|’s ≤ 0.26, all p’s ≥ 0.12).


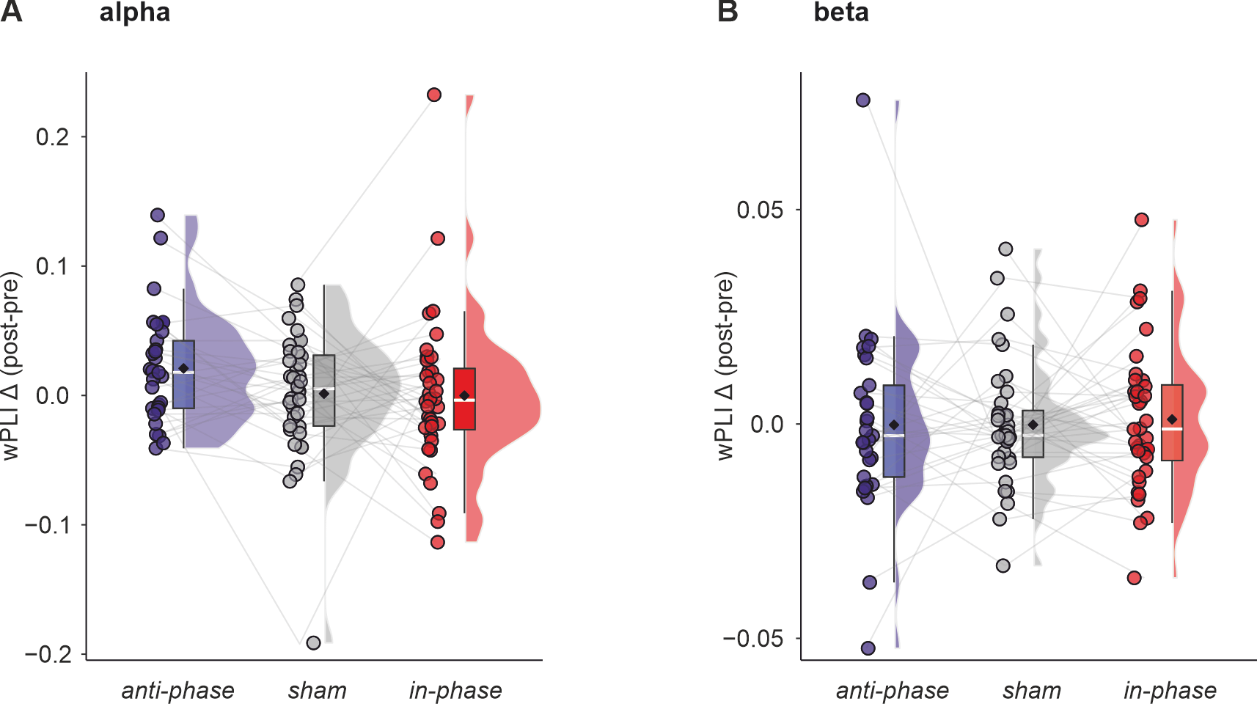


**Supplementary Figure 2. Connectivity.** (A) Fronto-parietal alpha and beta (B) connectivity (weighted phase lag index, wPLI). Raincloud plots show individual data points with the mean (diamond), and boxplots with median (white line), 25th and 75th percentiles (lower and upper hinges), and 1.5*interquartile range (lower and upper whiskers). Data points represent the difference between post and pre timepoints, subtracting “stim minus sham” in each individual. No modulation through stimulation was observed for alpha or beta connectivity (see Supplementary Table 3 for linear mixed model results). wPLI = debiased weighted phase-lag index. # of participants: 36, # of observations: 101.

**Supplementary Table 1.** Overview of preprocessing of included EEG data (n = 36).

|  | Sham | | In-phase | | Anti-phase | |
| --- | --- | --- | --- | --- | --- | --- |
|  | pre | post | pre | post | pre | post |
| n | 31 | 30 | 32 | 30 | 25 | 23 |
| Rejected channel mean (SD) | 3.87 (5.24) | 4.7  (5.19) | 3.75 (5.95) | 3.37 (4.75) | 3.84 (5.62) | 3.26 (4.23) |
| Rejected component mean (SD) | 2.52 (1.26) | 2.57 (1.07) | 2.59 (1.32) | 2.6  (1.07) | 2.44 (0.92) | 2.74 (1.25) |
| Rejected trials (%) mean (SD) | 5.23 (5.88) | 6.61 (6.36) | 6.96  (7.5) | 6.28 (5.25) | 6.01 (5.76) | 6.3  (7.09) |

*Note.* SD = standard deviation.

**Supplementary Table 2.** Model fit metrics and assumption tests for linear mixed-effects models.

|  |  |  | N obs.  (N subjects) | R² cond. | R² marg. | ICC | Linearity | Normality |
| --- | --- | --- | --- | --- | --- | --- | --- | --- |
| % correct | | | 119 (43) | 0.772 | 0.257 | 0.693 | ✓ | ✓ |
| wPLI | | |  |  |  |  |  |  |
|  | theta | | 101 (36) | 0.199 | 0.128 | 0.081 | ✓ | ✓ |
|  | alpha | | 101 (36) | 0.248 | 0.069 | 0.193 | ✓ | ✓ |
|  | beta | | 101 (36) | 0.276 | 0.050 | 0.237 | ✓ | ✓ |
| AECc | | |  |  |  |  |  |  |
|  | theta | | 101 (36) | 0.147 | 0.070 | 0.084 | ✓ | ✓ |
|  | alpha | | 101 (36) | 0.225 | 0.046 | 0.187 | ⚠ | ✓ |
|  | beta | | 101 (36) | NAᵃ | 0.011 | NAᵃ | ✓ | ✓ |
| Phase angles | | |  |  |  |  |  |  |
|  | theta | | 101 (36) | 0.174 | 0.074 | 0.108 | ✓ | ✓ |
|  | alpha | | 101 (36) | NAᵃ | 0.014 | NAᵃ | ✓ | ✓ |
|  | beta | | 101 (36) | NAᵃ | 0.110 | NAᵃ | ⚠ | ✓ |

*Note.* N obs. = number of observations (number of subjects in parentheses). R² cond. = conditional R² (total variance explained by fixed and random effects); R² marg. = marginal R² (variance explained by fixed effects only); ICC = intraclass correlation coefficient (proportion of variance attributable to between-subject clustering). Linearity: assessed via likelihood ratio test comparing linear versus natural spline models (df = 3) for age predictor; ✓ = linear relationship adequate (p > .05), ⚠ = non-linear relationship detected (p < .05). Normality: visual inspection of Q-Q plots for residuals and random effects. All models met normality assumptions via visual inspection. Independence of observations guaranteed by repeated-measures study design; random effects account for within-subject dependencies. ᵃ Singular model fit (random effect variance ≈ 0), indicating minimal between-subject variability for this measure. Marginal R² remains valid.

**Supplementary Table 3.** Estimated marginal means and pairwise comparisons from LMM analyses for alpha and beta connectivity change (wPLI).

|  |  |  | Estimate | Lower CI | Higher CI | *p*-value |
| --- | --- | --- | --- | --- | --- | --- |
| wPLI (alpha) | | |  |  |  |  |
|  | Marginal means (*10^-2^) | |  |  |  |  |
|  |  | Sham | 0.13 | -1.61 | 1.88 |  |
|  |  | Anti-phase | 2.18 | 0.24 | 4.11 |  |
|  |  | In-phase | 0.02 | -1.73 | 1.76 |  |
|  | Pairwise comparisons (*10^-2^) | |  |  |  |  |
|  |  | Anti-phase – sham | 2.04 | -0.34 | 4.43 | 0.092 |
|  |  | In-phase – sham | -0.12 | -2.35 | 2.12 | 0.918 |
|  |  |  |  |  |  |  |
| wPLI (beta) | | |  |  |  |  |
|  | Marginal means (*10^-2^) | |  |  |  |  |
|  |  | Sham | -0.01 | -0.60 | 0.57 |  |
|  |  | Anti-phase | -0.02 | -0.67 | 0.63 |  |
|  |  | In-phase | 0.11 | -0.47 | 0.70 |  |
|  | Pairwise comparisons (*10^-2^) | |  |  |  |  |
|  |  | Anti-phase – sham | -0.00 | -0.79 | 0.77 | 0.982 |
|  |  | In-phase – sham | 0.13 | -0.60 | 0.85 | 0.733 |


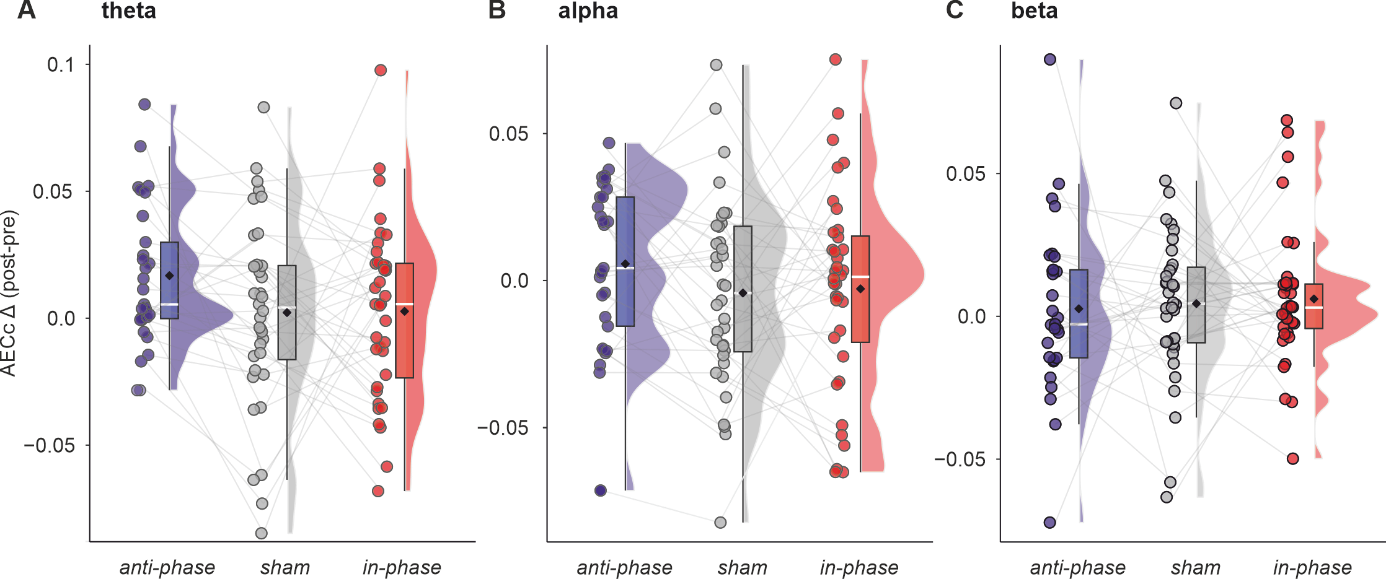


**Supplementary Figure 3. Amplitude envelope correlation.** (A) Fronto-parietal theta (A), alpha (B), and beta (C) connectivity. Raincloud plots show individual data points with the mean (diamond), and boxplots with median (white line), 25th and 75th percentiles (lower and upper hinges), and 1.5*interquartile range (lower and upper whiskers). Data points represent the difference between post and pre timepoints, subtracting “stim minus sham” in each individual. No modulation through stimulation was observed for alpha or beta connectivity (see Supplementary Table 4 for linear mixed model results). AECc = amplitude envelope correlation. # of participants: 36, # of observations: 101.


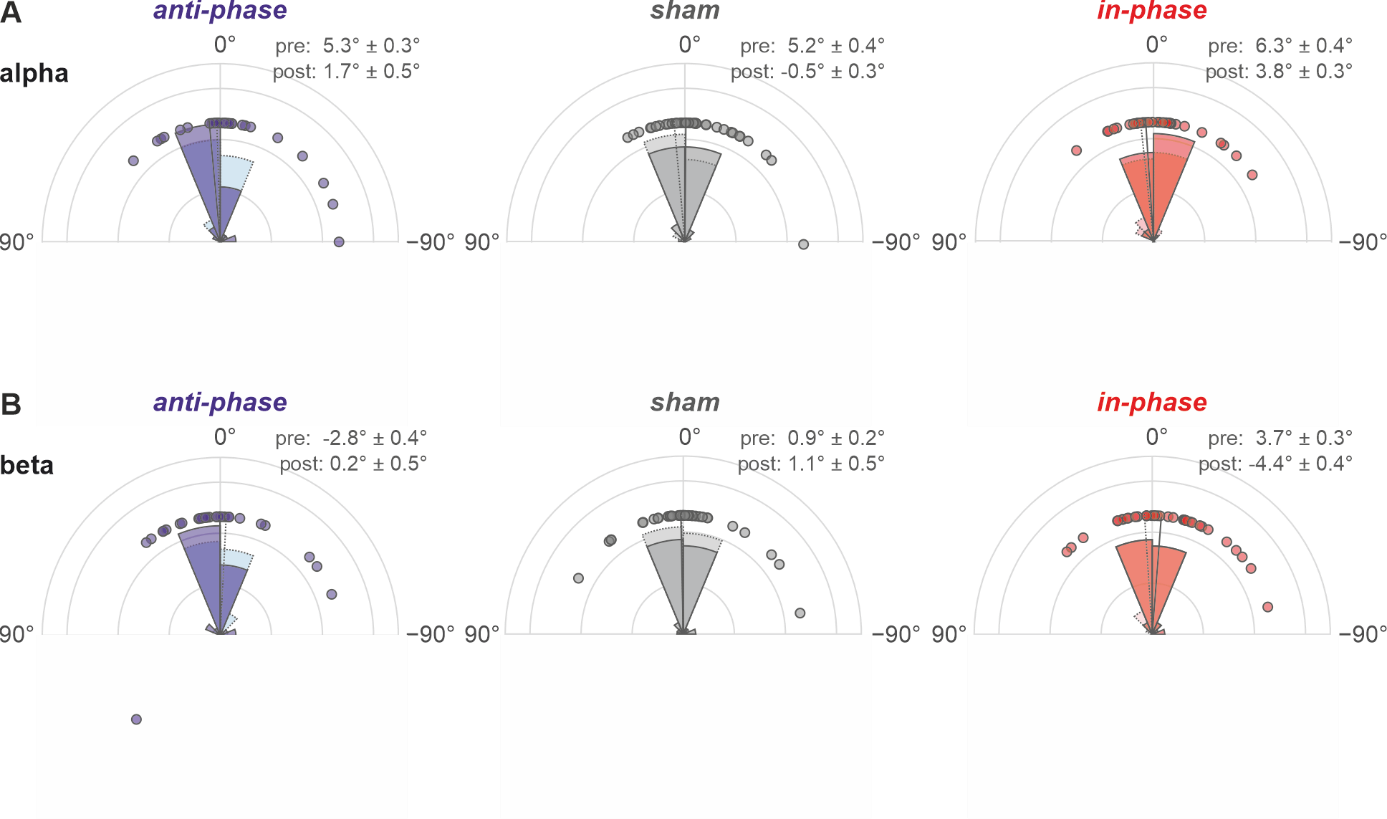


**Supplementary Figure 4. Alpha (A) and beta (B) phase angles.** Phase angle distributions (degrees) frequency shifts between stimulation targets and individual difference values between stimulation and sham conditions (blue dots). Histograms for pre (light blue) with mean vector (dashed line), and post (dark blue) with mean vector (solid line). No effects of stimulation conditions were observed for alpha (linear mixed models, all p’s > 0.2) and beta phase angles (linear mixed models, all p’s > 0.2). # of participants: 36, # of observations: 101.

**Supplementary Table 4.** Estimated marginal means and pairwise comparisons from LMM analyses for alpha and beta connectivity change (AECc).

|  |  |  | Estimate | Lower CI | Higher CI | *p*-value |
| --- | --- | --- | --- | --- | --- | --- |
| AECc (theta) | | |  |  |  |  |
|  | Marginal means (*10^-2^) | |  |  |  |  |
|  |  | Sham | 0.21 | -0.91 | 1.32 |  |
|  |  | Anti-phase | 1.68 | 0.43 | 2.92 |  |
|  |  | In-phase | 0.26 | -0.86 | 1.38 |  |
|  | Pairwise comparisons (*10^-2^) | |  |  |  |  |
|  |  | Anti-phase – sham | 1.47 | -0.15 | 3.09 | 0.074 |
|  |  | In-phase – sham | 0.05 | -1.47 | 1.57 | 0.944 |
| AECc (alpha) | |  |  |  |  |  |
|  | Marginal means (*10^-2^) | |  |  |  |  |
|  |  | Sham | -0.43 | -1.48 | 0.63 |  |
|  |  | Anti-phase | 0.51 | -0.66 | 1.69 |  |
|  |  | In-phase | -0.30 | -1.36 | 0.76 |  |
|  |  |  |  |  |  |  |
|  | Pairwise comparisons (*10^-2^) | |  |  |  |  |
|  |  | Anti-phase – sham | 0.94 | -0.51 | 2.39 | 0.199 |
|  |  | In-phase – sham | 0.13 | -1.23 | 1.48 | 0.853 |
| AECc (beta) | |  |  |  |  |  |
|  | Marginal means (*10^-2^) | |  |  |  |  |
|  |  | Sham | 0.45 | -0.45 | 1.35 |  |
|  |  | Anti-phase | 0.26 | -0.75 | 1.28 |  |
|  |  | In-phase | 0.60 | -0.30 | 1.51 |  |
|  | Pairwise comparisons (*10^-2^) | |  |  |  |  |
|  |  | Anti-phase – sham | -0.19 | -1.55 | 1.18 | 0.787 |
|  |  | In-phase – sham | 0.15 | -1.13 | 1.44 | 0.812 |

**Supplementary Table 5.** Adverse events by stimulation condition.

|  | Anti-phase | Sham | In-phase |
| --- | --- | --- | --- |
|  | Mean (SD) | | |
| Itching | 0.2 (0.4) | 0.1 (0.4) | 0.2 (0.5) |
| Pain | 0.1 (0.4) | 0 (0) | 0.1 (0.3) |
| Burning | 0.2 (0.6) | 0.2 (0.6) | 0.2 (0.4) |
| Warmth/heat | 0.1 (0.4) | 0.1 (0.4) | 0.1 (0.4) |
| Metallic taste | <0.1 (0.2) | 0 (0) | <0.1 (0.2) |
| Fatigue/decreased alertness | 0.4 (0.6) | 0.4 (0.7) | 0.3 (0.6) |
| Vibration/Pulsing | 0.1 (0.4) | 0.1 (0.4) | 0.1 (0.5) |
| Tingling | 0.7 (0.9) | 0.7 (0.8) | 0.6 (0.9) |
| Other | 0.1 (0.4) | 0.1 (0.3) | 0.1 (0.3) |

*Note.* Adverse events were assessed using a Likert scale ranging from no (0) to strong (3) intensity.

**Supplementary Table 6.** Blinding by stimulation condition.

| Stimulation condition | Guess [n (%)] | | |
| --- | --- | --- | --- |
|  | Active | Do not know | Sham |
| Active  Anti-phase  In-phase | 39 (33.3)  17 (14.5)  22 (18.8) | 33 (28.2)  15 (12.8)  18 (15.4) | 2 (1.7)  1 (0.9)  1 (0.9) |
| Sham | 19 (16.2) | 20 (17.1) | 4 (3.4) |
